# Comparison of research methods for functional characterization of insect olfactory receptors

**DOI:** 10.1038/srep32806

**Published:** 2016-09-16

**Authors:** Bing Wang, Yang Liu, Kang He, Guirong Wang

**Affiliations:** 1 State Key Laboratory for Biology of Plant Diseases and Insect Pests, Institute of Plant Protection, Chinese Academy of Agricultural Sciences, Beijing, China

## Abstract

Insect olfactory receptors (ORs) in the peripheral olfactory system play an important role detecting elements of information from the environment. At present, various approaches are used for deorphanizing of ORs in insect. In this study, we compared methods for functional analysis of ORs *in vitro* and *in vivo* taking the candidate pheromone receptor OR13 of *Helicoverpa assulta* (HassOR13) as the object of our experiments. We found that the natural system was more sensitive than those utilizing transgenic *Drosophila.* The two-electrode voltage-clamp recording is more suitable for functional screening of large numbers of ORs, while the *in vivo* transgenic *Drosophila* system could prove more accurate to further validate the function of a specific OR. We also found that, among the different solvents used to dissolve pheromones and odorants, hexane offered good reproducibility and high sensitivity. Finally, the function of ORs was indirectly confirmed in transgenic *Drosophila*, showing that odor-activation of ORs-expressing olfactory receptor neurons (ORNs) can mediate behavioral choices. In summary, our results compare advantages and drawbacks of different approaches, thus helping in the choice of the method most suitable, in each specific situation, for deorphanizing insect ORs.

The sense of smell in insects is of critical importance for every aspect of their life. Perception of odors and pheromones starts with detection of volatile molecules at the periphery of the sensory system, involving olfactory receptors (ORs) expressed on the membrane of olfactory receptor neurons (ORNs)^1^,^2^,^3^. Activation of ORs by odorants triggers generation of ORN action potentials, that converge to glomeruli of antennal lobes and eventually are integrated with other sensory inputs in the central nervous system (CNS)^4^,^5^. Since the first insect ORs from *Drosophila melanogaster* were identified using a bioinformatics-based approach^6^, a large amount of data on ORs has been accumulating from various insect taxa, thanks to recent simple and inexpensive methods of transcriptome sequencing^7^,^8^,^9^,^10^,^11^,^12^,^13^,^14^,^15^,^16^. Identification of the OR repertoire represents the first step towards understanding how the insect integrates and processes the huge diversity of chemical messages in the environment, originating from food, enemies and mates^17^. Consequentially, several methods for the functional characterization of insect ORs have been developed in which large numbers of biologically relevant odorants can be tested^18^,^19^,^20^,^21^,^22^,^23^,^24^,^25^,^26^,^27^.

Previous reports indicated that ORs cannot be properly folded when expressed in bacteria. Instead, they can be functionally characterized in eukaryotes, either *in vitro* or *in vivo*, along with well-established experimental strategies^19^,^28^,^29^,^30^,^31^,^32^,^33^,^34^,^35^,^36^,^37^,^38^. *In vitro*, heterologous expression systems, including *Xenopus* oocytes, have been adopted to probe the function of insect ORs^19^,^28^,^32^,^39^,^40^,^41^,^42^,^43^,^44^,^45^,^46^,^47^,^48^,^49^,^50^,^51^,^52^,^53^,^54^. The first member of *Drosophila* was deorphanized using the *Xenopus* oocytes and two-electrode voltage-clamp system, this still being the most common technique adopted for heterologous expression^28^. Generally, *Xenopus* oocytes are injected with cRNAs encoding a specific OR and the odorant receptor co-receptor (Orco). The presence of Orco significantly increases the sensitivity and the specificity of individual ORs^19^.

Heterologous expression of odorant receptors can be also conducted *in vivo* using transgenic *Drosophila* techniques, which include two main paradigms, the “empty neuron” system^29^ and the *Or67d*^GAL4^ knock-in system^33^. The *Drosophila* “empty neuron” system, originally constructed for the deorphanization of *Drosophila* ORs in 2003, is a combination of a GAL4 driver line under the Or22a promoter in the Δ *halo* background and a fly line with UAS–‘OR gene’. In that way, the “Favorite” OR gene is inserted next to UAS-promoter to be expressed in the “empty/mutant” ab3A (*basiconic sensilla*) neuron. Another *Or67d*^GAL4^ knock-in system generates mutant alleles in which the open reading frame of Or67d is replaced with GAL4 and introduces independent UAS –‘OR gene’ transgene insertions into the *Or67d*^GAL4^ line, which allows for expression of the OR in the unique ORN of the antennal *trichoid sensilla* (at1). With both systems, the technique of single sensillum recording (SSR) is used to monitor the electrophysiological responses of OSNs expressing the exogenous candidate OR. The recent literature shows that *in vivo Drosophila* expression systems are used by several research groups for functional identification of odorant receptors from other insect species, because of close similarities with their natural cellular environment^17^,^33^,^55^,^56^,^57^,^58^,^59^,^60^,^61^,^62^,^63^.

A large number of insect ORs have been functionally investigated using the above mentioned methods^17^,^18^,^19^,^23^,^24^,^25^,^26^,^27^,^28^,^29^,^30^,^31^,^55^,^56^,^58^,^59^,^64^,^65^,^66^,^67^,^68^,^69^,^70^,^71^,^72^. In this study we investigate the strengths of some currently used method used to deorphanize insect ORs. Specifically, we have performed functional analysis of HassOR13 using both heterologous *Xenopus* expression and *in vivo Or67d*^GAL4^ knock-in *Drosophila* transgenic systems. Further, we compared the results obtained via approaches with the performance of ORNs expressing HassOR13 in the moth. Previous reports have shown that odorant receptors are responsible for the specificity of ORNs, thus relating the performance of an odorant receptor to electrophysiological recordings. We have also verified the function of ORs at the behavioral level. Finally, we have compared the performance of different solvents for *in vivo* electrophysiological recording. Our results highlight advantages and the drawbacks of the two main approaches for OR functional characterization and provide information guidelines to select a suitable method to deorphanize insect ORs.

## Results

### Comparison between *in vitro* and *in vivo* protocols

**Figure 1:**
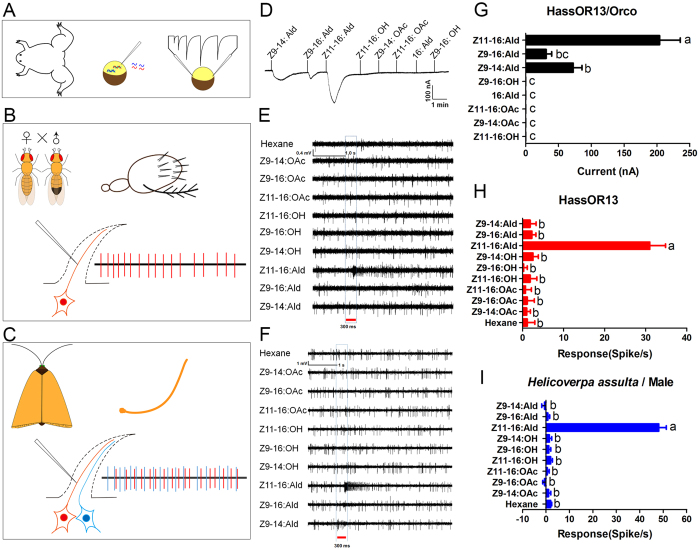
Functional comparison of HassOR13 responses between *in vitro* and *in vivo* methods. (**A**) Schematic diagram of *Xenopus* oocyte system. The morphological schematic of *Xenopus* is shown on the left and cRNA microinjection is shown in the middle. Responses of ORs were recorded using two-electrode voltage-clamp technique, as shown on the right. (**B**) Schematic diagram of *Or67d*^GAL4^ knock-in system. The morphological schematic of fly (male and female) is shown at the top on the left, fly antennae covered by the sensilla, mostly *trichoid sensilla* (at1) are shown at the top on the right, and responses of ORN from single sensilla (SSR) are shown at the bottom of the figure. (**C**) Schematic diagram of the endogenous system in moths. The morphological schematic of a moth is shown at the top left and its antennae at the top right. Responses of ORNs (SSR) are shown at the bottom of the figure. (**D**) Inward currents from HassOR13/HassOrco *Xenopus* oocytes in response to 10^−4^ M solutions of pheromone compounds. (**E**) SSR traces from HassOR13-expressing neurons in at1 *sensilla* of *Drosophila* in response to pheromone compounds. (**F**) SSR traces of HassOR13-expressing neurons in type A *sensilla* of *H. assulta* in response to pheromone compounds. (**G**) Response profile of HassOR13/HassOrco *Xenopus* oocytes. The amplitude evoked by Z11-16:Ald was significantly larger than others (*P* < 0.01, one-way ANOVA followed Duncan’s multiple range test, 204 ± 32 nA, n = 6). (**H**) Average SSR responses of HassOR13-expressing neurons in at1 *sensilla* of *Drosophila*. The response was exclusively elicited by Z11-16:Ald (*P* < 0.01, one-way ANOVA followed Duncan’s multiple range test, 31 ± 4 spikes ⁄ s, n = 7). (**I**) Average SSR responses of HassOR13-expressing neurons in type A *sensilla* of *H. assulta*. The response was exclusively elicited by Z11-16:Ald (*P* < 0.01, one-way ANOVA followed Duncan’s multiple range test, 48 ± 3 spikes ⁄ s, n = 9). Bars labelled with different letters are significantly different. Error bars indicate SEM.

**Figure 2:**
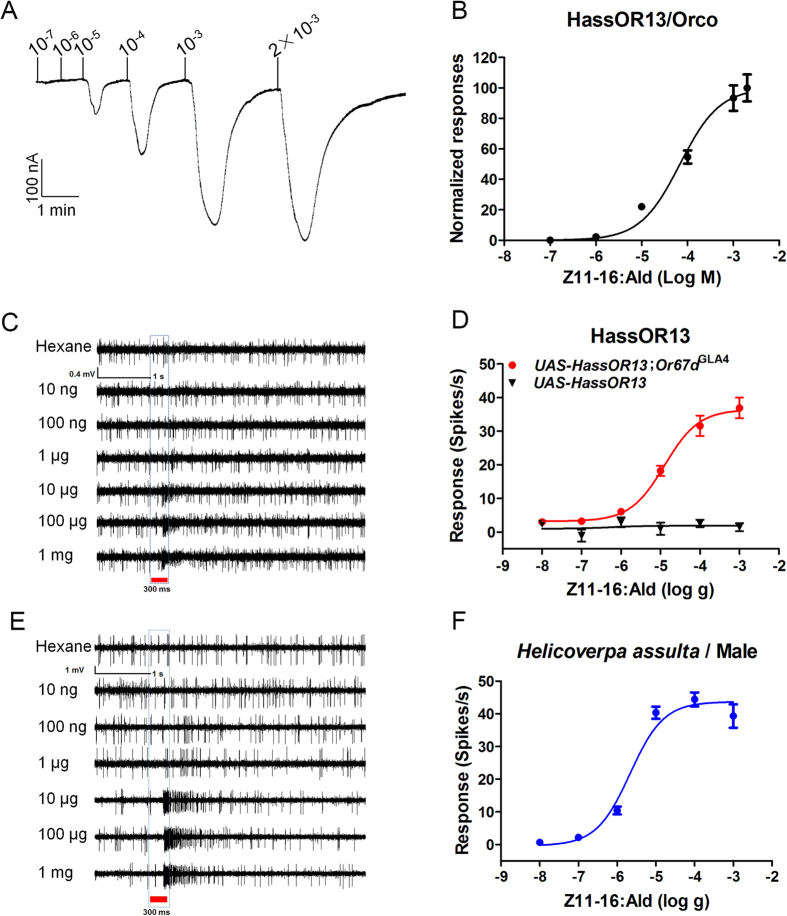
Dose-response relationships for *in vitro* and *in vivo* system. (**A**) HassOR13/HassOrco expressed in *Xenopus* oocytes and stimulated with a concentration range of Z11-16:Ald. (**B**) Dose–response curve of HassOR13/HassOrco stimulated with Z11-16:Ald across a series of concentrations from 10^−7^ M to 2 × 10^−3^ M (n = 7). Data are reported as percent of maximal responses. Error bars indicate SEM. (**C**) SSR traces showing Z11-16:Ald-evoked activity of at1 neurons from *UAS- HassOR13; Or67d*^GAL4^ homozygous line. No response was evoked by hexane. (**D**) Z11-16:Ald-induced dose–response curves from at1 neurons expressing (*UAS- HassOR13; Or67d*^GAL4^, n = 13) and non-expressing (*UAS-HassOR13*, n = 11) HassOR13, stimulated with 10^−3^ g to 10^−8^ g. Error bars indicate SEM. (**E**) SSR traces showing Z11-16:Ald-evoked neuronal activities in *H. assulta* across a range of concentrations. No response was evoked by hexane. (**F**) Z11-16:Ald-induced dose–response curves in *H. assulta* (n = 12). Error bars indicate SEM.

The receptor HassOR13 is tuned to the second sex pheromone component, (Z)-11-hexadecenal (Z11-16:Ald) in *H. assulta*^66^. Here, we used HassOR13 as a model to compare different methods of functional analysis. The first approach utilizes the *in vitro* heterologous expression systems in *Xenopus* oocytes (Fig. 1A). When co-expressed with Orco of *H. assulta* (HassOrco), HassOR13 robustly responded to Z11-16:Ald at a concentration of 10^−4^ M, but only weakly to the major pheromone component (Z)-9-hexadecenal (Z9-16:Ald) and to the non-specific pheromone (Z)-9-tetradecenal (Z9-14:Ald)^73^ (Fig. 1D). The signal evoked by Z11-16:Ald (204 ± 32 nA) was significantly larger than those produced by Z9-16:Ald and Z9-14:Ald, (72 ± 13 nA and 31 ± 8 nA, respectively, *P* < 0.01) (Fig. 1G). Dose–response experiments showed that the heterodimer HassOR13/HassOrco was sensitive to Z11-16:Ald with an EC_50_ value of 6.84 × 10^−5^ M (Fig. 2A,B and Table 1).

Using an *in vivo* system, the HassOR13 gene was expressed in at1 neurons of *Drosophila* and the resulting *UAS-HassOR13* flies were crossed with a mutant knock-in allele *Or67d*^GAL4^ driver line^33^. Then action potentials were recorded from the olfactory neurons within a single *sensillum* (Fig. 1B). The results showed that the HassOR13-expressing neurons in at1 specifically responded to the secondary sex pheromone component Z11-16:Ald at the dose of 1 mg loaded in the stimulus cartridge (*P* < 0.01) (Fig. 1E,H). In a dose–response experiment, neurons in at1 started firing at doses of Z11-16:Ald as low as 10 ng, with an EC_50_ value of 1.26 × 10^−5^ g (Fig. 2C,D, Table 1). For control lines *UAS-HassOR13*, no response to Z11-16:Ald was recorded at the same doses (Fig. 2D). We concluded that HassOR13 was selectively activated by Z11-16:Ald.

Recent *in situ* hybridization and single *sensillum* recording studies reported three types of *trichoid sensilla* on the antenna of *H. assulta*, with type A containing neurons responding only to Z11-16:Ald^66^,^74^. We directly recorded responses of *trichoid sensilla* type A from *H. assulta* antenna and compared the result with those obtained from transgenic fly lines (Fig. 1C). We first confirmed that neurons expressing HassOR13 gene were activated by Z11-16:Ald at a dose of 1 mg (*P* < 0.01) (Fig. 1F,I). Then, we measured the dose–response curve across a dose range from 10 ng to 1 mg (Fig. 2E,F) obtaining an EC_50_ value of 2.15 × 10^−6^ g (Table 1).

### Effect of solvent

**Figure 3:**
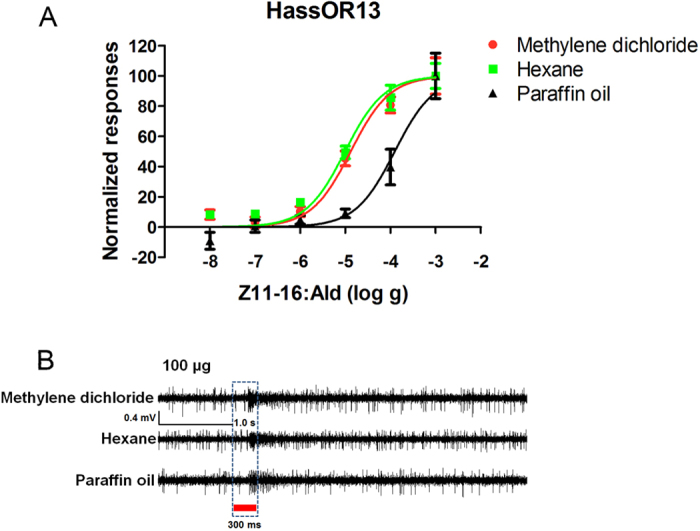
Functional comparison of HassOR13 expressed in *Drosophila* lines using different solvents. (**A**) Z11-16:Ald dose–response relationship for HassOR13 using paraffin oil, hexane or methylene dichloride as solvents. Z11-16:Ald was used at doses from 10^−3^ g to 10^−8^ g. Error bars indicate SEM. (**B**) SSR traces were recorded at the dose of 100 μg.

To evaluate the effects of different solvents used to dilute stimuli in single-sensillum experiments, we recorded responses of HassOR13 expressed in *Drosophila* at1 neurons to Z11-16:Ald dissolved in paraffin oil, hexane or methylene dichloride in a dose range from 10 ng to 1 mg (Fig. 3A). When the ligand was diluted in paraffin oil, the sensitivity (EC_50_ = 1.06 × 10^−4^ g) of HassOR13 to Z11-16:Ald in the system was markedly lower than when using methylene dichloride (EC_50_ = 1.31 × 10^−5^ g) or hexane (EC_50_ = 9.84 × 10^−6^ g) (Table 2). Figure 3 shows representative traces recorded at doses of 100 μg of the pheromone dissolved in the different solvents (Fig. 3B).

### *Drosophila* lines expressing OR13 are attracted to Z11-16:Ald

**Figure 4:**
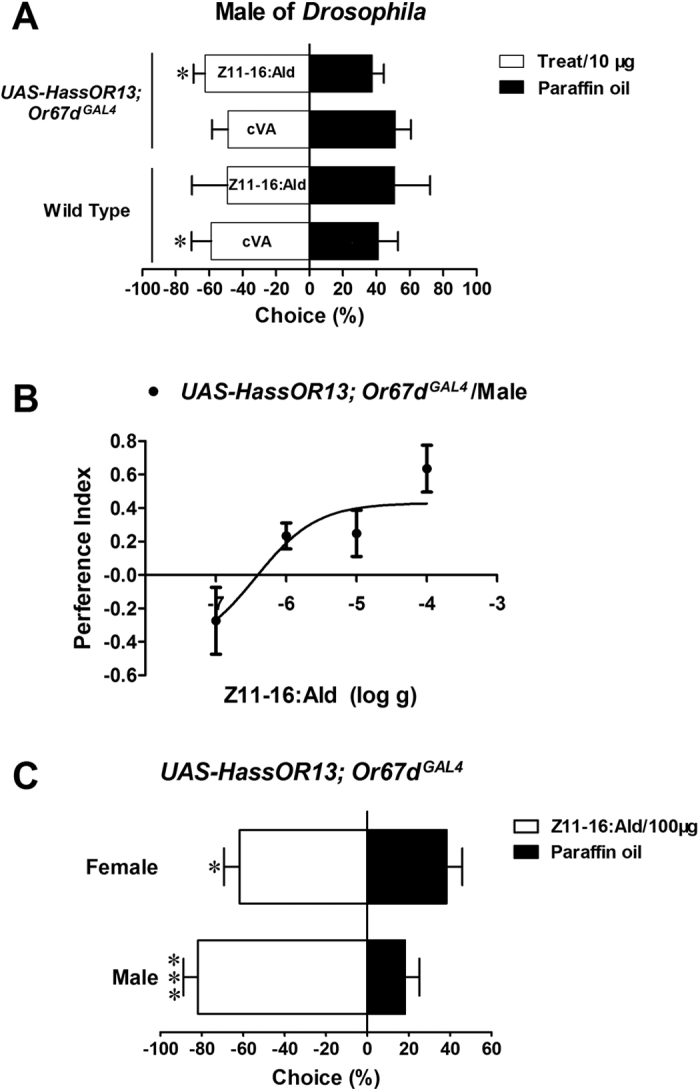
Attraction to Z11-16:Ald of *Drosophila* lines expressing HassOR13. (**A**) Two-choice behavioral assay at the dose of 10 μg. Wild-type flies display strong attraction toward cVA (*P < 0.05, Chi-square test, χ^2^ = 5.59, *df* = 1), which is abolished in HassOR13-expressing fly lines (*P* = 0.55, Chi-square test, χ^2^ = 0.35, *df* = 1). The attraction preference of HassOR13-expressing fly lines (*P < 0.05, Chi-square test, χ^2^ = 5.77, *df* = 1) and wild-type flies (*P* = 0.56, Chi-square test, χ^2^ = 0.34, *df* = 1) toward Z11-16:Ald are shown. Error bars indicate SEM. (**B**) Attraction of HassOR13-expressing male fly lines to Z11-16:Ald gradually increased with the dose of pheromone up to 10^−4^ g (n = 3~6). (**C**) Two-choice behavioral assay of HassOR13-expressing fly lines at the dose of 100 μg. Both males and females were significantly attracted to Z11-16:Ald compared to paraffin oil (for male, ***P < 0.001, Chi-square test, χ^2^ = 49.87, *df* = 1; for female, *P < 0.05, Chi-square test, χ^2^ = 6.22, *df* = 1). Error bars indicate SEM.

Next, we asked if HassOR13-expressing *Drosophila* would also exhibit a behavioral phenotype. Therefore, we performed behavior experiments using a two-choice bait trap assay^62^. Wild-type flies showed significant preference for 11-cis-vaccenyl acetate (cVA) compared to Z11-16:Ald and to paraffin oil (*P* < 0.05) (Fig. 4A). However, flies expressing HassOR13 were attracted to Z11-16:Ald (*P* < 0.05), but not to cVA (Fig. 4A) at a dose of 10 μg. In *UAS-HassOR13; Or67d*^GAL4^ lines, attraction to Z11-16:Ald was observed at doses from 10^−7^ g to 10^−4^ g. The attraction preference index (PI) of male flies gradually increased with the amount of Z11-16:Ald up to 10^−4^ g with an EC_50_ value of 3.7 × 10^−7^ g (Fig. 4B). In these experiments both male and female transgenic flies were attracted to the moth pheromone (Fig. 4C). Taken together, these data indicate that HassOR13 can mediate attraction to Z11-16:Ald in *Drosophila* by activating at1 neurons, thus confirming the function of this odorant receptor.

## Discussion

Rapidly and accurately deorphanizing OR genes is very important to elucidate how the insect converts external chemical signals into electrical signals through ORNs at the periphery of the olfactory system. Among the several methods developed during the last decade for the functional characterization of insect ORs, including the use of transgenic *Drosophila* and heterologous expression in *Xenopus* oocytes, it is sometimes difficult to choose the most suitable protocol for each research purpose. In this study we have compared *in vitro* and *in vivo* systems to study the function of HassOR13. In both cases, cells or neurons expressing HassOR13 were specifically activated by Z11-16:Ald. Of the two *in vivo* approaches, we found that the endogenous system was more sensitive (EC_50_ = 2.15 × 10^−6^ g) than that utilizing transgenic *Drosophila* (EC_50_ = 1.26 × 10^−5^ g). A comparison between *in vivo* and *in vitro* systems is not feasible, because we record electric currents in the two-electrode voltage-clamp technique used for heterologous expression systems, while we measure frequency of firing (spikes⁄s) when recording from single *sensilla* of transgenic flies. Each method presents its advantages and drawbacks. Sometimes, OR function cannot be properly reproduced in *Xenopus* expression system probably due to the absence of odorant binding proteins (OBPs)^65^,^71^. On the other hand, OR genes cannot always be expressed in transgenic fly lines^17^. Therefore, *in vitro* or *in vivo* protocols must be adopted depending on specific requirements. For example, the two-electrode voltage clamp recording is more practical in functional screenings of large numbers of ORs, while the *in vivo* transgenic *Drosophila* system is generally more accurate.

As for the choice of a solvent to dissolve odorant stimuli, we tested the three most used in the literature, paraffin oil, methylene dichloride and hexane^20^,^55^,^56^,^58^. The last two provided stronger responses compared to paraffin oil. This is due to the much lower volatility of the pheromone when dissolved in paraffin oil. Hexane remains probably the solvent of choice, offering a good reproducibility with a high sensitivity, while methylene dichloride can generate spontaneous firing of a specific neuron in some cases (Figure S1). However, paraffin oil has its advantage when testing a large number of odorants, since highly volatile compounds are likely to evaporate less from this solvent.

In our study, we used the *Or67d*^GAL4^ knock-in system to express HassOR13 gene in at1 *sensilla* of *Drosophila*. On the basis of the one-to-one relationship between ORs and ORNs, as well as on the odor-selective activation of ORs, we tested behavioral responses of transgenic fly lines to odorants. In general, *Orco*^GAL4^ driven UAS-OR lines or the lines with odorant receptor promoter to drive expression of GAL4 have been adopted to monitor behavioral preference for specific odors matching ORs-expressing neurons within defined *sensilla*^36^,^37^,^62^. In our research we performed behavioral assays with flies expressing HassOR13 in their ORNs under control of the *Or67d*^GAL4^ driver background. We observed strong attraction of both male and female transgenic lines to Z11-16:Ald with a EC_50_ value in agreement with single sensillum recording. This indicates that odor-activation of ORs-expressing ORNs can mediate behavioral choice. At the same time, we can use behaviour assays to indirectly identify the function of specific ORs, without the need to perform electrophysiological recordings.

In summary, we have compared different approaches to study the function of ORs *in vitro* and *in vivo*, presenting the advantages and the drawbacks of each method. Studying the interactions of pheromones and odorants with their receptors still requires complex methodologies, as ORs cannot be expressed and isolated in their active forms.

## Materials and Methods

### Insect rearing

*H. assulta* individuals were reared in our laboratory with an artificial diet at the larval stage^75^ and 10% honey solution at the adult stage, at 26 ± 1 °C, 65% ± 5% relative humidity and under photoperiod of 16 h light: 8 h dark. Pupae were sexed and put into separate cages for eclosion. *Drosophila* stocks were fed on cornmeal-agar-molasses medium and maintained under a 12 h light: 12 h dark cycle at 25 °C and 60% relative humidity.

### Pheromone components

(Z)-9-hexadecenol-1-ol (Z9-16:OH), (Z)-9-tetradecen-1-ol (Z9-14:OH), (Z)-9-tetradecenyl acetate (Z9-14:OAc), (Z)-9-hexadecadecenyl acetate (Z9-16:OAc), (Z)-11-hexadecenal (Z11-16:Ald) and (Z)-11-hexadecen-1-ol (Z11-16:OH) (both 95% minimum purity) were purchased from Nimrod Inc. (Changzhou, China). (Z)-9-tetradecenal (Z9-14:Ald), (Z)-9-hexadecenal (Z9-16:Ald), (Z)-11-hexadecenyl acetate (Z11-16:OAc) (all 93–95% minimum purity) were purchased from Bedoukian (Danbury, CT, USA). Paraffin oil, methylene chloride and hexane (96–98% minimum purity) were purchased from Sigma-Aldrich Co. (St. Louis, MO, USA).

### cRNA synthesis and Electrophysiological recording with two-electrode voltage-clamp

HassOR13 and HassOrco genes were cloned into eukaryotic expression vector pT7Ts and stored as plasmids in our laboratory. cRNAs were synthesized using mMESSAGE mMACHINE T7 Ultra Kit (Ambion, Austin, TX, USA) following the manufacturer’s instructions. HassOR13 was expressed in *Xenopus* oocytes according to the following protocol. 27.6 ng of both HassOR13 and HassOrco cRNA were microinjected into mature oocytes (stage V–VII), that had been treated with 2 mg/mL collagenase in washing buffer (96 mM NaCl, 2 mM KCl, 5 mM MgCl_2_, and 5 mM HEPES, pH 7.6) for 1–2 h at room temperature. Then, oocytes were cultured for 4–7 days at 18 °C in 1 × Ringer’s solution (96 mM NaCl, 2 mM KCl, 5 mM MgCl_2_, 0.8 mM CaCl_2_, and 5 mM HEPES, pH 7.6) supplemented with 5% dialysed horse serum, 50 mg/mL tetracycline, 100 mg/mL streptomycin and 550 mg/mL sodium pyruvate. The recording methods of two-electrode voltage-clamp followed previously reported protocols^25^,^27^. Whole-cell currents were obtained from the injected *Xenopus* oocytes with a two-electrode voltage-clamp and recorded with an OC-725C oocyte clamp (Warner Instruments, Hamden, CT, USA) at a holding potential of −80 mV. Oocytes were exposed to compounds in ascending order of concentration with an interval between exposures that allowed the current to return to baseline. Data acquisition and analysis were carried out with Digidata 1440 A and PCLAMP 10.2 software (Axon Instruments Inc., Union City, CA, USA). GRAPHPAD PRISM 5.0 software (GraphPad Software Inc., San Diego, CA, USA) was used to analyze dose–response data.

### Fly strains

Transgenic lines were generated according to standard procedures as described below. The open reading frame encoding HassOR13 was cloned into the pVALIUM20 vector^76^. Independent homozygous *UAS-HassOR13* lines (with transgene insertions into chromosome II) were generated at Tsinghua Fly Center (Beijing, China). Driver mutant allele *Or67d*^*GAL4*^ stock was provided by Dr Barry J. Dickson^33^. The balancer *w−/w−; sp/CyO; TM3/TM6B* was used to cross with homozygous driver lines. Then, the driver line in *Or67d*^*GAL4*^ mutant background was crossed with *UAS-HassOR13* balancer line to establish final homozygous stock *w+/w+; UAS-HassOR13/UAS-HassOR13; *Or67d*^GAL4^/*Or67d*^GAL4^* which expressed HassOR13 in at1 *sensilla* neurons. Each HassOR13 insertion was confirmed by sequencing of genomic DNA prepared from mutant lines. Both final stock and wild-type Canton-S strain were used for electrophysiological experiments.

### Single sensillum recordings

Extracellular electrophysiological recordings were performed on single at1 *sensilla* of 1- to 10-day-old flies. The antenna was fixed using standard procedures^55^,^77^. The reference electrode was placed in the fly eye, under a microscope (LEICA Z16 APO, Germany) at 920 × magnification. Action potentials were recorded by inserting a tungsten wire electrode in the base or in the shaft of a *sensillum* of the fly antenna. Signals were amplified 10 × by a high impedance pre-amplifier (IDAC-4 USB System, Syntech, Kirchzarten, Germany), sent to a PC via an analog-digital converter and analyzed off-line with AUTOSPIKE v. 3.9 software (Syntech, Kirchzarten, Germany). The filter was set with a 500 Hz low cutoff and a 3 kHz high cutoff. AC signals were recorded for 10 s, starting 1 s before stimulation. Responses were calculated by counting the number of action potentials one second after stimulation (with a delay of 200 ms to allow the odorant to travel down the airstream), and subtracting the number counted in the second before stimultion.

### Odor stimulation

Aliquots of odorants were dissolved in paraffin oil, methylene chloride or hexane (vol/vol) and 10 μL of each solution were loaded onto a 0.5 × 40 mm filter paper strip (Whatman), which was placed inside a Pasteur pipette. Hexane, methylene chloride or paraffin oil alone were tested as negative controls. For dose-response relationships, serial dilutions were made in increasing doses of 0.001, 0.01, 0.1, 1, 10 and 100 μg/μL and loaded on filter paper strips. The preparation was held in a humidified continuous air flow delivered by the Syntech Stimulus controller (CS-55 model, Syntech) at 1.4 L/min. Stimulus pulses were added for 300 ms. During stimulation, the compensatory flow was switched off.

### Behavioral assays

Attraction to odours was measured using a modified two choice trap assay^62^,^78^. Two to three day old female and male (1:1) adult fruit flies were starved for 40–42 h in collection cages containing 1% agarose gel. 40–60 flies per repeat were anaesthetized on ice, then placed into a 1 L glass beaker covered with a 150 mm Petri dish with three holes covered by nylon mesh for ventilation. Odor traps were made from 40 mL plastic vials with a 1 mL pipette tip inserted at the top, and placed in the glass beaker. Traps contained a filter paper strip soaked with either 10 μL of the odor (cVA or Z11-16:Ald) at different dilutions in paraffin oil or just paraffin oil was added. Behaviour tests were conducted for 24 h in the dark at room temperature. Each treatment was repeated three to six times. Dose–response curves (10^−7^ to 10^−4^) were used to calculate the preference index (PI), according to the formula PI = (#flies in odor vial − #flies in control vial)/total # of flies.

### Statistical analysis

All data were presented as mean ± SEM. Data multiple comparison over three groups was assessed by one-way analysis of variance (ANOVA) following Duncan’s multiple range test for variable (*α* = 0.05), and two-sample analysis was performed using Student’s *t*-test (*α* = 0.05). Two choice trap assay results were compared using Chi-square test. All statistic comparison were assayed with SPSS Statistics 16.0 (SPSS Inc., Chicago, IL, USA).

## Additional Information

**How to cite this article**: Wang, B. *et al*. Comparison of research methods for functional characterization of insect olfactory receptors. *Sci. Rep.* **6**, 32806; doi: 10.1038/srep32806 (2016).

## Supplementary Material

Supplementary Information

## Figures and Tables

**Table 1:** Comparison of dose-response values recorded with *in vitro* and *in vivo* system.

Comparison in different systems		EC_50_ value	Standard error of EC_50_	Test number
*In vitro*	*Xenopus* oocyte system	6.84 × 10^−5^ M	7.93 × 10^−6^	7
*In vivo*	Transgenic system in *Drosophila*	1.26 × 10^−5^ g	5.47 × 10^−6^	13
	The endogenous system in *H. assulta*	2.15 × 10^−6^ g	4.10 × 10^−7^	12

**Table 2:** Dose-response values in HassOR13-expressed in *Drosophila* lines using different solvents.

*Or67d*^GAL4^ knock-in system, *in vivo*		EC_50_ value	Standard error of EC_50_	Test number
Different solvents	Methylene dichloride	1. 31 × 10^−5^ g	1.10 × 10^−5^	12
	Hexane	9.84 × 10^−6^ g	5.47 × 10^−6^	13
	Paraffin oil	1.06 × 10^−4^ g	6.92 × 10^−5^	10
