# Supplementary material for: Comparison of research methods for functional characterization of insect olfactory receptors
Source: Sci Rep. 2016 Sep 16;6:32806. doi: 10.1038/srep32806 (PMC5025650; doi:10.1038/srep32806)
Supplement: Supplementary Information [file srep32806-s1.pdf]

Comparison of research methods for functional characterization of insect olfactory receptors

Bing Wang, Yang Liu, Kang He, Guirong Wang*

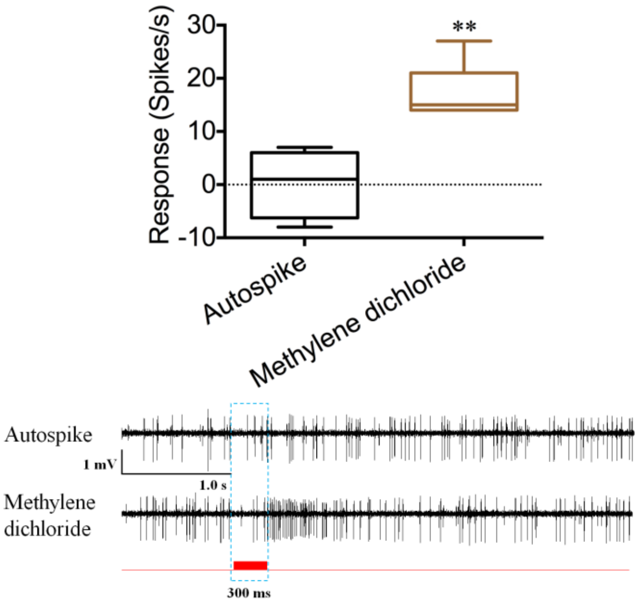

Figure S1. Responses activated by methylene dichloride in *Drosophila melanogaster* lines. SSR traces showing methylene dichloride-evoked activity (**$P < 0.01$, Student's t-test, 18 ± 2 spikes∕s, n =4–7). Error bars indicate SEM.
